# Dosimetric comparison of 3D-conformal radiotherapy, volumetric modulated arc therapy, and h-VMAT in left breast radiotherapy under free-breathing and breath-hold conditions

**DOI:** 10.1038/s41598-025-16665-3

**Published:** 2025-09-26

**Authors:** Shambhavi C, Sarath S. Nair, Jyothi Nagesh, Anshul Singh, Umesh Velu, Ankita Mehta, N. P. Jayashree, Mahak Gupta, Jayapalan Krishnan, Kathiresan K, Vennila J, Shirley Lewis, Krishna Sharan

**Affiliations:** 1 Medical Radiation Physics Program, Department of Radiation Oncology, Manipal College of Health Professions , Manipal Academy of Higher Education, Manipal, Karnataka 576104 India; 2 Department of Radiation Oncology, Kasturba Medical College, Manipal, Manipal Academy of Higher Education, Manipal, Karnataka 576104 India; 3 Department of Radiation Oncology, Yenepoya Medical College Hospital, Deralakatte, Mangalore, Karnataka 575018 India; 4 Department of Radiation Oncology, VPS Lakeshore Hospital & Research Centre, Ernakulam, Cochin, Kerala 682040 India; 5 Manipal College of Health Professions, Manipal Academy of Higher Education, Manipal, Karnataka 576104 India; 6 Department of Radiotherapy and Oncology, Justice K. S. Hegde Medical College, NITTE, Mangalore, Karnataka 575018 India

**Keywords:** Hybrid, h-VMAT, Left breast, Radiotherapy, DIBH, Cancer, Health care, Oncology

## Abstract

This study investigates the role of the h-VMAT (a hybrid of 3DCRT and VMAT) technique in the Deep Inspirational Breath-Hold (DIBH) method for left-breast irradiation by comparing dosimetric parameters of 3DCRT, VMAT, and h-VMAT plans in free breathing and breath-hold conditions. The study enrolled fourteen left breast cancer patients with nodal involvement. Five sets of plans, namely, 3DCRT, VMAT, and three different dose ratios of h-VMAT, were created in both the DIBH and Free-Breathing (FB) data sets. All plans had similar planning criteria and were compared using Dose-Volume parameters of the target and critical organs. Both h-VMAT and VMAT achieved acceptable PTV parameters compared to 3DCRT. A significant reduction in heart mean dose was observed in the h-VMAT80/20 (*p* < 0.001) under both DIBH and FB methods and was found to be minimal compared to 3DCRT and VMAT. Similarly, Left Anterior Descending Artery (LAD) dose was also reduced in hybrid. Additionally, the left lung volume receiving 20 Gy and 30 Gy were less in hybrid. We conclude that h-VMAT, along with DIBH, is successful in achieving the best trade-off between heart exposed to high and low doses of radiation compared to 3DCRT and VMAT, respectively, without compromising target coverage for left breast radiotherapy.

## Introduction

Breast cancer is one of the most diagnosed cancers in women^1^. Based on GLOBOCAN figures from 2020, breast cancer accounts for 11.7% of all other cancers and is the fifth leading cause of cancer-related deaths globally^2^. Adjuvant Radiation Therapy (RT) for conservative breasts in early-stage breast cancer and mastectomy in locally advanced breast cancer is a standard of care treatment and, hence, significant in improving local recurrence, and long-term survival rates^3,4^. Consequently, a good quality of life can be provided by ensuring reduced doses of nearby critical organs, and it is also necessary to consider the long-term effects of RT to avoid secondary cancers. The ipsilateral lung is one of the major Organs at Risk (OAR) in breast or chest wall RT; however, heart poses additional importance in left breast cancer. Review on long-term follow-up randomized trials reported radiation toxicities to the lungs and heart, involves second lung cancer and radiation-induced cardiac mortality, with estimated excess rate ratios (ERR) of 0.11 and 0.04 per Gy for whole organ, respectively^5^. It is evident from previous studies that radiation-induced cardiac complications increased linearly with heart mean dose^6^. Hence, it is essential to reduce the volume of OARs being irradiated. In the recent development of RT, Deep Inspiration Breath Hold (DIBH) is a respiratory control method used for organ motion management that plays a vital role in left breast RT. As the patient holds a deep breath, the lungs fill with air, displacing the heart away from the chest wall and, hence, from the target volume. Studies have shown that the DIBH technique has significantly spared the heart dose^7^as well as lung dose^8^ and certain anatomical predictors are reported to help identify patients who would be benefited^9^.

Through the decades, considerable evolutions happened in RT of breast cancer such as the schedule of dose fractionation and the treatment techniques. The conventional dose schedule has been replaced with a 3-week schedule of 42.6 Gy–40.05 Gy in 16 or 15 fractions, respectively, which has been accepted worldwide^10–12^. According to the treatment planning point of view, breast RT has always been considered a challenging task due to the complex anatomical region. Three-dimensional conformal radiotherapy(3DCRT) of wedged tangential beams, intensity-modulated RT (IMRT) of multiple static fields, and volumetric modulated arc therapy (VMAT) are the various standard treatment techniques used in the current era. Even though 3D-CRT technique is widely available, cost-effective, and involves simple beam arrangements for treatment planning, it has associated disadvantages that are of concern^13^ especially, the inadequate target coverage at the field junction of the Supra-Clavicular Fossa (SCF) and breast or chest wall. Moreover, it results in high doses of healthy tissue within the RT portals. Conversely, implementing inverse-planning techniques, like IMRT or VMAT, improves target dose coverage, homogeneity, and conformity and reduces the high doses to critical organs compared to 3D-CRT techniques^14,15^. Nevertheless, some studies have also speculated a higher possibility of secondary malignancies in critical organs due to the larger low dose received volume of the same organs^16^. Considering both the pros and cons of static field and modulated field techniques, Mayo et al. introduced a special technique called hybrid-IMRT by combining 3D-CRT and IMRT techniques for whole breast RT^15^. It is evident from the literature that hybrid techniques for breast RT successfully enhance target dose conformity and reduce OAR doses^18,19^.

Most of the previous breast RT research articles have investigated the benefits of h-VMAT, a hybrid of the 3DCRT and VMAT planning techniques^20–22^. However, to the best of our knowledge, a study comparing h-VMAT planning under Free Breathing (FB) condition and breath-hold specialized technique for hypo-fractionated RT in both whole breast and chest wall irradiation has not been well studied. To investigate whether combining h-VMAT and DIBH techniques has any additional benefits regarding plan quality, this dosimetric study aims to compare the 3DCRT, VMAT, and the various combinations of h-VMAT planning techniques under FB and DIBH method for hypo-fractionated left breast RT by evaluating Planning Target Volume (PTV) and OAR dose parameters.

## Materials and methods

### Patient selection

The study was conducted according to the STROBE guidelines. Ethical approval was granted by the KMC & KH Institutional Ethics Committee (IEC No: 837/2021) and prospectively registered with the Clinical Trials Registry – India (CTRI/2021/08/035673). Fourteen left breast cancer female patients after Breast Conservative Surgery (BCS) or mastectomy, with nodal involvement who had received a hypo-fractionated RT dose of 40.05 Gy in 15 fractions by h-VMAT technique under breath-hold condition at our center during the year 2023 to 2024 were enrolled. The DIBH and FB image sets of the patients were replanned in this retrospective study.

### Simulation and volume delineation

All patients were immobilized on a breast board and positioned supine with arms over their heads. After each patient practiced adequately for the breath-holding method using the Active Breathing controller tool (ABC, Elekta), two sets of planning computed tomography (CT) scans of 3 mm slice thickness were acquired, one during DIBH and another in FB condition on the Philips Brilliance Big Bore 16-slice CT. These CT scan images were then exported to MONACO (version 5.11, M/s Elekta Ltd, UK) treatment planning system (TPS) for contouring and treatment planning. The RTOG (Radiation Therapy Oncology Group) and ESTRO (European Society for Radiotherapy and Oncology) breast contouring guidelines were referred for delineating target volume and OARs^23^. Clinical Target Volume (CTV) involves breast or chest-wall for all patients with the inclusion of regional nodes. A uniform margin of 5 mm around CTV was added to generate PTV in both DIBH, and also in FB image sets to maintain uniformity in planning comparison. OAR delineation included heart, lungs, right breast, esophagus, and spine.

### Treatment planning

**Fig. 1:**
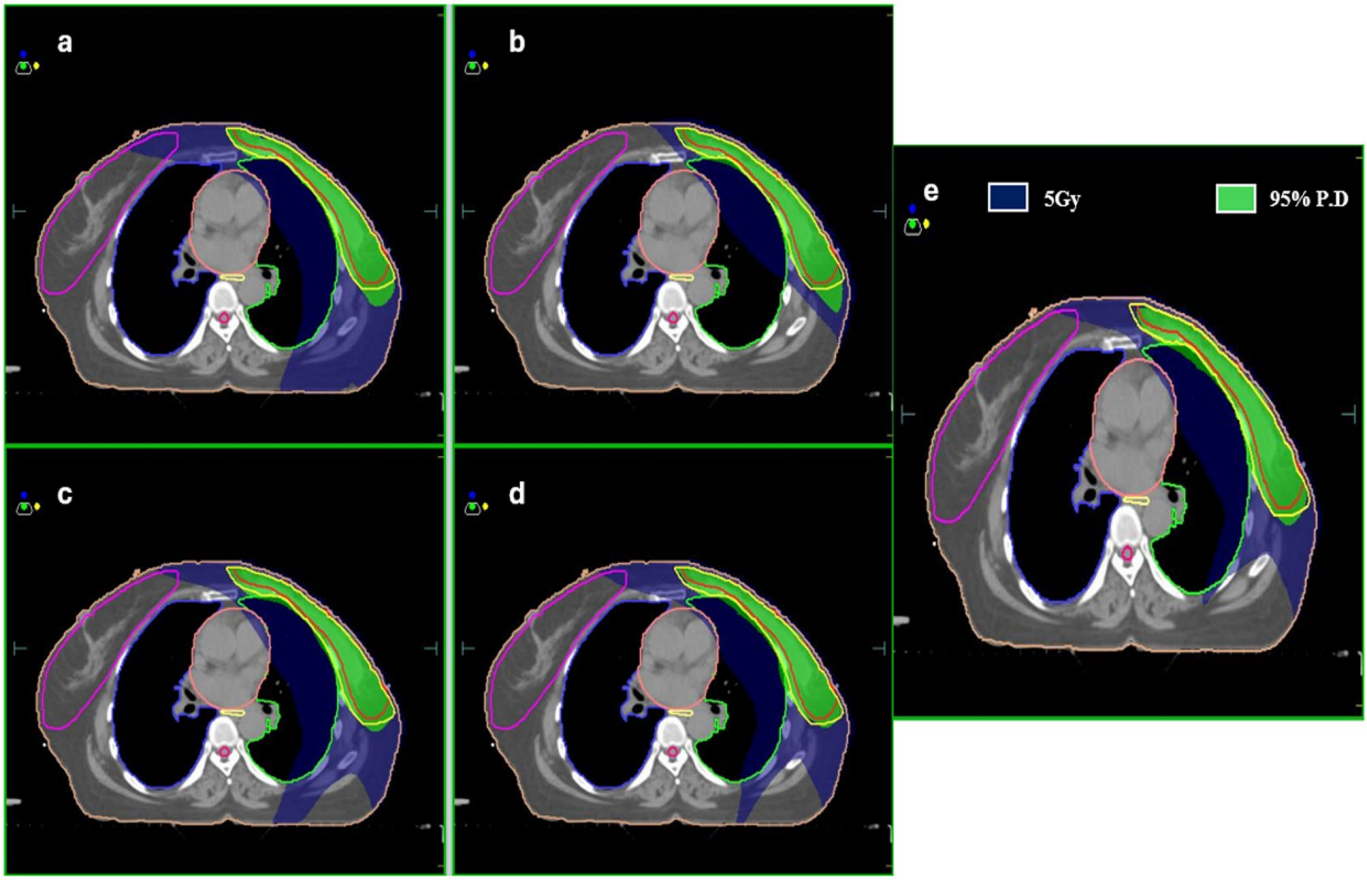
Isodose distribution in color-wash representing 95% P.D. and 5 Gy for VMAT (**a**), 3DCRT (**b**), h-VMAT60/40 (**c**), h-VMAT70/30 (**d**) and h-VMAT80/20 (**e**) plans under the DIBH method.

The planning objectives for left breast RT with a prescription dose (PD) of 40.05 Gy in 15 fractions to PTV were as per the departmental norms. For tumor: PTV receiving 95% of the PD ≥ 95%, and a hotspot 107% PD of < 2 cc was accepted. Under OAR objectives, the heart was given a higher priority, with mean dose < 2 Gy, volume receiving 10 Gy (V10Gy) < 3% and V25Gy < 10%. Other constraints included, left lung: V12Gy < 30%, V20Gy < 20%; right breast: mean dose < 2 Gy and right lung: mean dose < 2 Gy. According to the planning objectives, five sets of plans were generated in both DIBH and FB image sets, namely 3DCRT, VMAT, and three different combinations of h-VMAT (h-VMAT80/20, h-VMAT70/30 and h-VMAT60/40) for Elekta VERSA HD linear accelerator. Plan isocenter was placed at the junction of supraclavicular field (SCF) and breast/chest wall. and kept identical for all plans to avoid planning bias. Plan description as follows:

3DCRT in DIBH & FB: Associated with mono-isocentric conformal half-blocked fields of a single-direct anterior-supraclavicular and two opposed wedged tangential using 6MV (medially) and 10MV (laterally) photons (Fig. 1b). Tangential gantry angles were chosen between 300 and 310 and 120 to 130 degrees to achieve maximum sparing of the heart and other organs. Both SCF and tangential fields were normalized midway through the fields, and the dose was calculated using the Collapsed Cone Convolution algorithm with a 3 mm dose calculation grid. Hotspots were reduced by copying the main tangential beams (without wedge) of reduced dose ratio and blocking them with multi-leaf collimators (MLC) by visualizing in beams eye view (BEV).

VMAT in DIBH & FB: Generated using two coplanar partial arcs of 6MV photons with an arc span of 200^0^ to 220^0^ as shown in Fig. 1a, by setting minimum segment width to 5 mm and control points to 200. The Monte Carlo algorithm was used for plan optimization in IMRT constraint mode with a 3 mm dose calculation grid size. The cost function constraints for OARs and PTV were chosen according to planning objectives.

h-VMAT in DIBH & FB: Generated by combining 3DCRT field-in-field (FinF) and the VMAT plans as shown in Fig. 1e. The 3DCRT-FinF plan was made with the same geometrical parameters as that of the 3DCRT plan. However, wedges were removed from the tangential fields, and 2 to 3 MLC segments were added to get uniform dose distribution. Three different h-VMAT plans were made: h-VMAT 80/20, h-VMAT 70/30, and h-VMAT 60/40. In the h-VMAT 80/20 plan, the 3DCRT-FinF was a base plan, which contributed 80% of prescribed dose, and the remaining 20% contributed by VMAT to provide for dose-optimization. Similarly, h-VMAT 70/30 (Fig. 1d) and h-VMAT 60/40 (Fig. 1c) plans were created by copying the h-VMAT 80/20 plan and changing the corresponding dose ratio of 3DCRT-FinF and VMAT plans before dose calculation.

### Plan evaluation

As per the planning objectives, the Dose Volume Histogram (DVH) parameters were analysed to find the dose received by PTV and OARs. Further, PTV dose Conformity Index (CI) and Homogeneity Index (HI) were calculated as follows:

1 $${\text{CI}}=\left( {{\text{TV95}}\% /{\text{V95}}\% } \right) \times \left( {{\text{V95}}\% /{\text{TV}}} \right)$$

2 $${\text{HI}}=\left( {{\text{D2}}\% - {\text{D98}}\% } \right)/{\text{D5}}0\%$$

where TV is the target volume, V95% is 95% isoline volume, TV95% is PTV volume receiving 95% isodose, and Dx% represents the minimum dose received by x% of PTV.

Plan Quality Index (PQI) was an additional formula adopted in this study to provide scoring for each plan based on plan merit and to select a better plan^24^.

3 $${\text{Plan Quality Index }}\left( {{\text{PQI}}} \right)={\text{ID}}{{\text{I}}_{{\text{Tumor}}}} \times {\text{ID}}{{\text{I}}_{{\text{Organs}}}}$$

where IDI_Tumor_ is the Integrated Dosimetry Index score for tumor or PTV, and IDI_Organs_ is the Integrated Dosimetry Index score for organs. Here, HI (Ideal value is zero) and CI (Ideal value is one) are two indices used for calculating the Integrated Dosimetry Index score for tumor. The IDI_Organs_ score was calculated by using OAR planning dose constraints and achieved dose constraints, wherein V10Gy and V25Gy for the heart, V12Gy and V20Gy for the left lung, and mean dose for the right breast and right lung were included. The target and organ dose-volume parameters used for calculating PQI score were given equal weightage and a plan that gets the lowest PQI score was considered the best.

### Statistical analysis

The mean and standard deviation of the normally distributed data were calculated and taken for statistical analysis in Jamovi Software (Version 2.3.28). A paired t-test was used to compare dosimetric differences between plans of the same technique generated in two different imaging methods (FB and DIBH), and a repeated measures ANOVA statistical test was performed to compare all five plans generated in the DIBH method, with *p* < 0.05 was assumed to be statistically significant.

## Results

For all the five types of plans generated, an initial comparison was made between DIBH and FB methods of the same technique. DIBH results were comparatively favorable in all the techniques in reducing OAR doses, and hence, the next comparison was made among all five planning techniques under the DIBH methods keeping the treated plan as a standard plan as described in the following.

### Plan comparison under FB vs. DIBH method

**Fig. 2:**
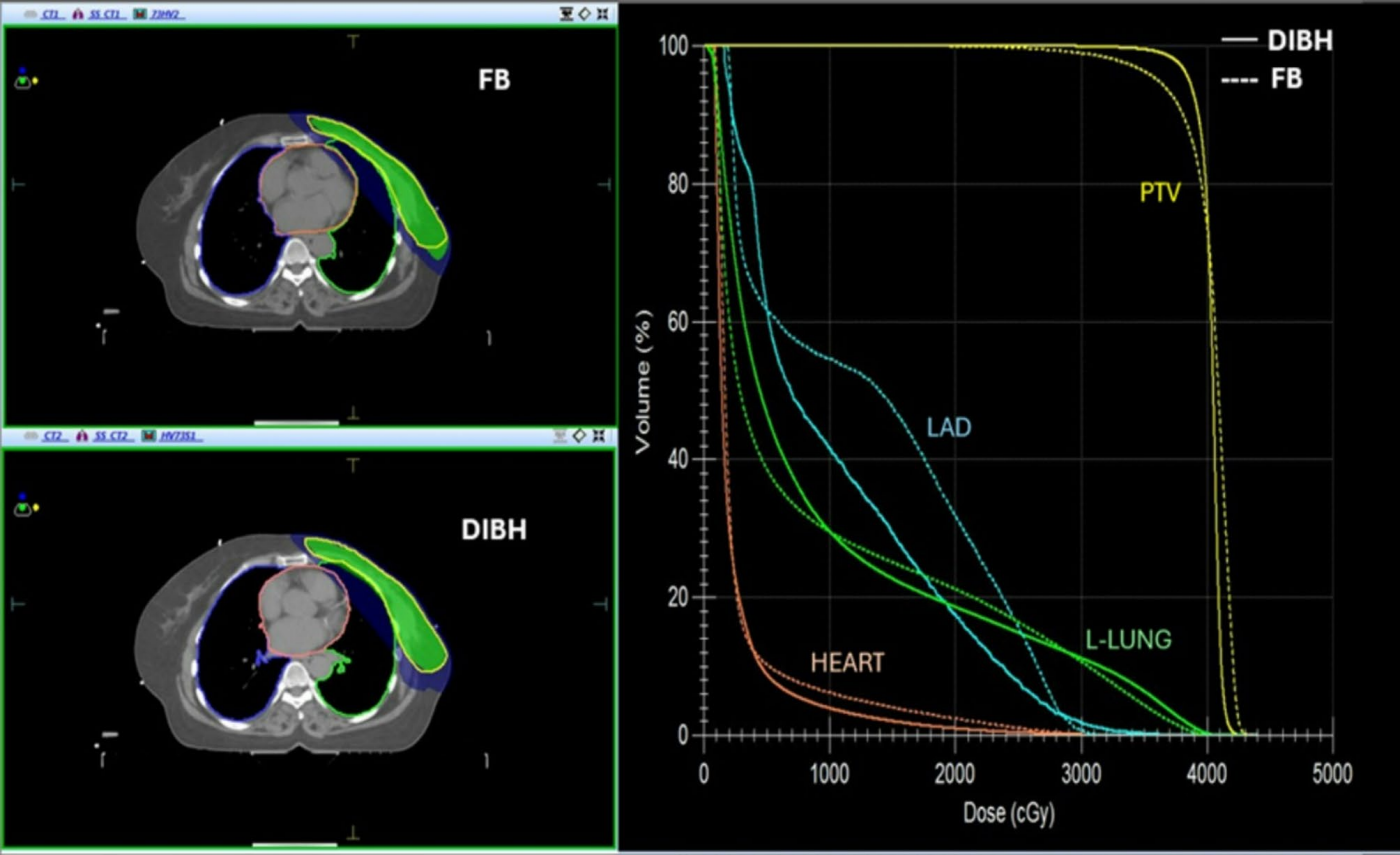
Comparison of dose distribution and DVH parameters for a case under DIBH and FB conditions.

Figure 2 illustrates the dose distribution and DVH parameters of a selected planning technique under both methods for a case and their dosimetric results are summarized in Tables 1 and 2. There was no significant difference between the FB and DIBH methods in the PTV parameters of all the plans except for VMAT and h-VMAT60/40(Table 1). However, VMAT showed better PTV coverage in DIBH and FB (97.05 ± 1.51 Vs. 95.66 ± 1.41, *p* = 0.017) and h-VMAT60/40 showed a good PTV coverage of 96.72 ± 1.77 in FB. In addition, a significant difference was observed in the CI of h-VMAT80/20 (*p* = 0.032) and HI of VMAT(*p* = 0.016) plans between the two methods. Table 2 shows the OAR parameters for both methods. Under DIBH, noticeable statistical differences with *p* < 0.01 were seen in all the dosimetric parameters of the heart for all plans, where in mean dose was significantly reduced by 1 Gy compared to FB. Further, a significance reduction of LAD mean, and maximum dose were observed in DIBH. Similarly, various dose-volume parameters of the left lung, including mean dose, V30Gy, V12Gy, and V5Gy, were comparatively less in the DIBH method except for V5Gy in h-VMAT and VMAT plans. Moreover, the left lung mean dose showed a significant reduction with DIBH (*p* < 0.05) for all plans, with a maximum reduction in 3DCRT compared to other plans (1.5 Gy Vs. 0.4 Gy). Esophagus maximum dose was comparatively low in the DIBH method for h-VMAT 80/20 (36.75 ± 2.87 Gy, *p* = 0.04), and 3DCRT (33.98 ± 4.40 Gy, *p* = 0.022) plans. Overall, both methods observed no reduction in mean dose for the contralateral side breast and lung.

**Table 1:** Comparison of PTV dosimetric matrices between FB and DIBH for all planning techniques: paired t-test, P-value < 0.05; SD = standard deviation; HI = homogeneity index; CI = conformity index.

Technique	PTV dosimetric parameters	Method (Mean ± SD)		p value
		DIBH	FB	
h-VMAT 80/20	PTV-SCF (95%)	97.33 ± 2.40	97.34 ± 3.15	0.994
	PTV-CW (95%)	95.54 ± 1.89	96.24 ± 1.73	0.36
	TotalPTV95 (%)	95.61 ± 1.53	95.52 ± 1.82	0.863
	HI	0.13 ± 0.04	0.13 ± 0.02	0.751
	CI	0.72 ± 0.08	0.75 ± 0.07	**0.032**
h-VMAT 70/30	PTV-SCF (95%)	97.42 ± 2.23	98.14 ± 1.71	0.274
	PTV-CW (95%)	95.98 ± 1.96	96.70 ± 1.47	0.23
	TotalPTV95 (%)	95.79 ± 1.74	96.13 ± 1.40	0.491
	HI	0.13 ± 0.05	0.13 ± 0.02	0.754
	CI	0.73 ± 0.08	0.74 ± 0.06	0.571
h-VMAT 60/40	PTV-SCF (95%)	98.5 ± 1.32	98.18 ± 2.18	0.596
	PTV-CW (95%)	95.23 ± 1.76	96.72 ± 1.77	**0.027**
	TotalPTV95 (%)	95.64 ± 1.87	96.13 ± 1.90	0.439
	HI	0.14 ± 0.04	0.13 ± 0.02	0.492
	CI	0.71 ± 0.09	0.73 ± 0.07	0.347
3DCRT	PTV-SCF (95%)	74.24 ± 7.63	77.93 ± 9.44	0.275
	PTV-CW (95%)	91.19 ± 4.77	90.18 ± 5.77	0.36
	TotalPTV95 (%)	89.43 ± 4.78	87.88 ± 5.38	0.126
	HI	0.19 ± 0.04	0.18 ± 0.03	0.332
	CI	0.58 ± 0.15	0.56 ± 0.14	0.221
VMAT	PTV-SCF (95%)	98.51 ± 1.36	98.50 ± 1.24	0.996
	PTV-CW (95%)	96.85 ± 1.60	96.43 ± 1.56	0.537
	TotalPTV95 (%)	97.05 ± 1.51	95.66 ± 1.41	**0.017**
	HI	0.11 ± 0.04	0.13 ± 0.02	**0.016**
	CI	0.75 ± 0.09	0.75 ± 0.07	0.83

**Table 2:** Represents the OAR matrices for FB and DIBH methods of all planning techniques: paired t-test, P-value < 0.05; (M ± S) = Mean ± Standard deviation; VxGy(%)=%volume reviecing xGy; R-Lung = Right lung; R-Breast = Right breast; LAD = left anterior descending artery; Esophagus-Max = Esophagus maximum dose.

Technique	Left Lung					Heart						LAD		*R*-Lung	*R*-Breast	Esophagus
	Mean (Gy)	V20Gy (%)	V30Gy (%)	V12Gy (%)	V5Gy (%)	Mean (Gy)	Max (Gy)	V25Gy (%)	V5Gy (%)	V10Gy (%)	V2Gy (%)	Mean (Gy)	Max (Gy	Mean (Gy)	Mean (Gy)	Max (Gy)
h-VMAT80/20																
DIBH (M ± S)	9.83 ± 0.78	18.51 ± 2.93	9.97 ± 2.06	27.00 ± 2.78	48.67 ± 6.54	2.76 ± 0.86	33.67 ± 7.31	1.34 ± 1.69	8.92 ± 4.33	4.67 ± 3.23	31.66 ± 8.73	12.92 ± 6.40	28.89 ± 9.79	1.45 ± 0.38	1.66 ± 0.54	36.75 ± 2.87
FB (M ± S)	10.51 ± 0.71	22.78 ± 3.71	12.14 ± 1.81	29.81 ± 3.67	46.56 ± 3.30	3.82 ± 0.83	37.84 ± 2.0	3.70 ± 2.19	12.89 ± 3.35	8.40 ± 3.26	39.04 ± 6.58	18.84 ± 5.90	35.34 ± 7.58	1.45 ± 0.26	1.67 ± 0.34	38.55 ± 1.84
p	**0.003**	**< 0.001**	**< 0.001**	**0.006**	0.234	**< 0.001**	**0.036**	**< 0.001**	**0.001**	**< 0.001**	**0.005**	**0.004**	**0.02**	0.956	0.9	**0.04**
h-VMAT70/30																
DIBH (M ± S)	9.97 ± 0.68	18.28 ± 2.80	9.70 ± 1.80	27.54 ± 1.99	49.72 ± 5.16	2.86 ± 0.76	33.76 ± 7.48	1.30 ± 1.46	9.40 ± 3.69	5.13 ± 3.26	33.08 ± 6.89	13.23 ± 6.11	29.66 ± 9.75	1.62 ± 0.38	1.90 ± 0.72	36.84 ± 2.66
FB (M ± S)	10.58 ± 0.76	22.35 ± 3.45	11.78 ± 1.52	30.14 ± 3.51	47.87 ± 5.05	3.93 ± 0.80	38.14 ± 2.37	3.53 ± 1.97	13.99 ± 3.59	8.94 ± 2.97	40.82 ± 8.39	18.89 ± 5.58	35.68 ± 7.65	1.71 ± 0.33	1.94 ± 0.36	36.68 ± 9.66
p	**0.005**	**< 0.001**	**< 0.001**	**0.02**	0.154	**< 0.001**	**0.026**	**< 0.001**	**< 0.001**	**< 0.001**	**< 0.001**	**0.002**	**0.017**	0.409	0.812	0.953
h-VMAT60/40																
DIBH (M ± S)	10.49 ± 0.91	19.52 ± 3.07	10.45 ± 2.34	30.37 ± 3.0	51.65 ± 5.58	3.12 ± 0.79	34.14 ± 7.03	1.39 ± 1.50	11.54 ± 4.40	6.39 ± 3.04	35.77 ± 9.46	14.19 ± 6.17	30.17 ± 9.53	1.75 ± 0.39	1.98 ± 0.59	37.78 ± 2.50
FB (M ± S)	10.90 ± 0.80	22.02 ± 4.79	12.17 ± 2.05	31.46 ± 2.97	49.92 ± 4.70	4.02 ± 0.62	38.12 ± 2.15	3.26 ± 1.54	14.89 ± 3.13	9.30 ± 2.66	44.19 ± 5.99	18.68 ± 5.36	36.01 ± 6.74	1.80 ± 0.34	2.13 ± 0.42	36.90 ± 9.75
p	**0.033**	**0.012**	**0.03**	0.123	0.107	**< 0.001**	**0.028**	**< 0.001**	**0.009**	**0.002**	**0.008**	**0.012**	**0.012**	0.661	0.336	0.714
3DCRT																
DIBH (M ± S)	9.12 ± 1.66	20.48 ± 5.00	13.28 ± 3.97	26.00 ± 5.32	36.82 ± 5.32	3.08 ± 1.33	37.4 ± 7.21	3.62 ± 2.89	10.45 ± 5.36	7.26 ± 4.42	22.63 ± 6.90	17.88 ± 7.46	35.14 ± 7.63	0.42 ± 0.11	0.77 ± 0.50	33.98 ± 4.40
FB (M ± S)	10.69 ± 1.44	25.06 ± 4.40	16.58 ± 3.64	31.05 ± 4.56	41.65 ± 4.32	4.11 ± 1.41	40.48 ± 1.04	5.91 ± 3.26	14.21 ± 4.97	10.58 ± 4.32	27.01 ± 5.99	22.68 ± 6.44	39.02 ± 4.51	0.46 ± 0.06	0.70 ± 0.22	36.96 ± 1.56
p	**< 0.001**	**< 0.001**	**0.001**	**< 0.001**	**0.002**	**< 0.001**	0.123	**< 0.001**	**0.002**	**0.008**	**< 0.001**	**0.015**	0.108	0.15	0.464	**0.022**
VMAT																
DIBH (M ± S)	10.95 ± 0.78	20.59 ± 2.34	10.20 ± 1.93	33.26 ± 2.43	54.77 ± 6.40	3.59 ± 0.68	34.15 ± 5.13	1.45 ± 1.29	13.78 ± 3.09	7.21 ± 2.65	50.42 ± 10.14	14.06 ± 4.63	30.86 ± 6.90	2.49 ± 0.65	2.67 ± 0.85	38.35 ± 3.17
FB (M ± S)	11.4 ± 0.53	22.13 ± 1.79	10.90 ± 1.68	34.06 ± 2.80	55.68 ± 4.79	4.40 ± 0.63	37.27 ± 3.60	2.68 ± 1.91	17.67 ± 3.43	9.70 ± 2.90	63.08 ± 11.58	17.57 ± 4.47	33.89 ± 5.92	2.69 ± 0.86	2.99 ± 0.72	38.88 ± 2.57
p	**0.046**	0.052	0.298	0.24	0.551	**< 0.001**	**0.005**	**0.007**	**< 0.001**	**< 0.001**	**0.009**	**0.032**	**0.009**	0.191	0.152	0.576

### Plans comparison under the DIBH method

**Fig. 3:**
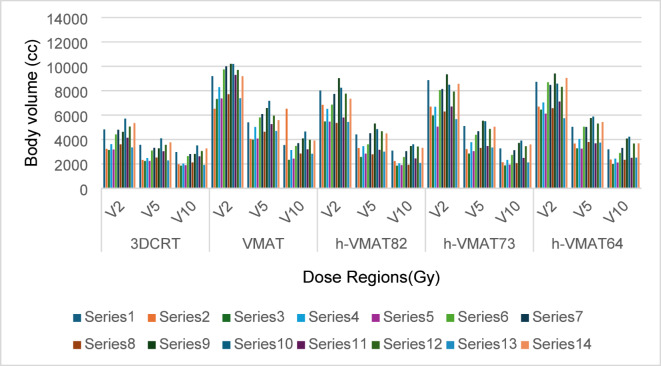
Comparison of body segments receiving low-dose bath of 2 Gy, 5 Gy and 10 Gy among all the techniques.

As shown in Fig. 1 isodose distribution ranges from 5 Gy to 44 Gy for a selected case under breath-hold conditions. Here the conformal dose coverage of 95% PD to PTV was observed for VMAT and hybrid plans, compared to the 3DCRT plan, which showed a higher spread of dose coverage outside the PTV. The 5 Gy isodose area was shown to account for low dose spill, which looks spread over a larger area in VMAT in comparison with 3DCRT hybrid plans. In addition, it is observed from graphical representation (Fig. 3) of body segments receiving low dose spill of 2 Gy, 5 Gy and 10 Gy are considerably higher in VMAT than 3DCRT (> 50%) and hybrid (> 30%). Although the 2 Gy dose spill were seen higher in hybrid plans (50%) than 3DCRT, 5 Gy (28%) and 10 Gy (4%) spill were almost comparable. To find a better-quality plan, various dosimetric matrices for PTV were analyzed, as listed in Table 3. According to that, the VMAT showed a higher dose coverage to Total PTV, PTVSCF, and PTV-CW/BR by nearly 97% followed by hybrid plans with approximately 95%. However, the dose coverage by 3DCRT were 89.43 ± 4.78%, 74.24 ± 7.63%, and 91.19 ± 4.77% respectively, with *p* < 0.05 compared to both h-VMAT80/20 and VMAT. In addition, 3DCRT resulted in inferior HI (0.19 ± 0.04) and CI (0.58 ± 0.15) as they deviate much from the ideal value compared to h-VMAT80/20 (HI = 0.13 ± 0.04; CI = 0.72 ± 0.08), VMAT (HI = 0.11 ± 0.04; CI = 0.75 ± 0.09) with *p* < 0.05. No significant difference was found among the three ratios of hybrid plans for PTV matrices.

**Table 3:** Comparison of dosimetric parameters of all planning techniques in the DIBH method: repeated measures ANOVA test, P-value < 0.05; SD = standard deviation; IDI_tumor_ = integrated dosimetry index for tumor; IDI_organs_ = integrated dosimetry index for organ;  PQI= plan quality index; MU = monitor unit; (Mean = Median; SD = IQR/1.35).

Structure	Dosimetric parameter	Technique(Mean ± SD)					*p*-value				
		h-VMAT 80/20(a)	h-VMAT 70/30(b)	h-VMAT 60/40(c)	3DCRT (d)	VAMT(e)	a Vs b	a Vs c	a Vs d	a Vs e	d Vs e
PTV	SCF 95% (%)	97.33 ± 2.40	97.42 ± 2.23	98.5 ± 1.32	74.24 ± 7.63	98.51 ± 1.36	1.000	0.243	**< 0.001**	0.500	**< 0.001**
	CW/BR95%(%)	95.54 ± 1.89	95.98 ± 1.96	95.23 ± 1.76	91.19 ± 4.77	96.85 ± 1.60	0.940	0.971	**0.01**	0.164	**0.002**
	TotalPTV95% (%)	95.61 ± 1.53	95.79 ± 1.74	95.64 ± 1.87	89.43 ± 4.78	97.05 ± 1.51	0.990	1.000	**< 0.001**	**0.031**	**< 0.001**
	HI	0.13 ± 0.04	0.13 ± 0.05	0.14 ± 0.04	0.19 ± 0.04	0.11 ± 0.04	0.999	0.777	**< 0.001**	0.354	**0.001**
	CI	0.72 ± 0.08	0.73 ± 0.08	0.71 ± 0.09	0.58 ± 0.15	0.75 ± 0.09	0.089	0.998	**0.001**	0.487	**< 0.001**
IDI_Tumor_		8.94 ± 2.94	8.68 ± 3.33	9.25 ± 3.47	15.48 ± 6.02	7.24 ± 2.16	0.605	0.781	**0.001**	0.244	**0.001**
Left Lung	Mean (Gy)	9.83 ± 0.78	9.97 ± 0.68	10.49 ± 0.91	9.12 ± 1.66	10.95 ± 0.78	0.970	**0.045**	0.529	**0.009**	**0.007**
	V20Gy (%)	18.51 ± 2.93	18.28 ± 2.80	19.52 ± 3.07	20.48 ± 5.00	20.59 ± 2.34	0.970	0.143	0.203	0.206	1.000
	V30Gy (%)	9.97 ± 2.06	9.70 ± 1.80	10.45 ± 2.34	13.28 ± 3.97	10.20 ± 1.93	0.882	0.383	**0.008**	0.994	0.085
	V12Gy (%)	27.00 ± 2.78	27.54 ± 1.99	30.37 ± 3.0	26.00 ± 5.32	33.26 ± 2.43	0.954	0.009	0.937	**< 0.001**	**< 0.001**
	V10Gy (%)	30.89 ± 2.70	31.57 ± 2.13	34.70 ± 3.31	27.93 ± 5.44	37.46 ± 3.09	0.962	**0.014**	0.393	**< 0.001**	**< 0.001**
	V5Gy (%)	48.67 ± 6.54	49.72 ± 5.16	51.65 ± 5.58	36.82 ± 5.32	54.77 ± 6.40	0.962	0.396	**0.01**	0.098	**< 0.001**
Heart	Mean (Gy)	2.76 ± 0.86	2.86 ± 0.76	3.12 ± 0.79	3.08 ± 1.33	3.59 ± 0.68	0.934	**0.044**	0.694	**0.008**	0.477
	Max (Gy)	33.67 ± 7.31	33.76 ± 7.48	34.14 ± 7.03	37.4 ± 7.21	34.15 ± 5.13	0.999	0.940	**0.021**	0.994	**0.036**
	V25Gy (%)	1.34 ± 1.69	1.30 ± 1.46	1.39 ± 1.50	3.62 ± 2.89	1.45 ± 1.29	0.995	0.984	**0.012**	0.999	**0.038**
	V5Gy (%)	8.92 ± 4.33	9.40 ± 3.69	11.54 ± 4.40	10.45 ± 5.36	13.78 ± 3.09	0.981	**0.044**	0.587	**0.006**	0.121
	V10Gy (%)	4.67 ± 3.23	5.13 ± 3.26	6.39 ± 3.04	7.26 ± 4.42	7.21 ± 2.65	0.867	**0.022**	0.055	**0.019**	1.000
	V2Gy (%)	31.66 ± 8.73	33.08 ± 6.89	35.77 ± 9.46	22.63 ± 6.90	50.42 ± 10.14	0.887	0.081	**0.007**	**< 0.001**	**< 0.001**
LAD	Max (Gy)	28.89 ± 9.79	29.66 ± 9.75	30.17 ± 9.53	35.14 ± 7.63	30.86 ± 6.90	0.545	0.478	**0.024**	0.775	0.099
	Mean (Gy)	12.92 ± 6.40	13.23 ± 6.11	14.19 ± 6.17	17.88 ± 7.46	14.06 ± 4.63	0.956	0.067	**0.002**	0.587	0.185
R-Lung	Mean (Gy)	1.45 ± 0.38	1.62 ± 0.38	1.75 ± 0.39	0.42 ± 0.11	2.49 ± 0.65	0.095	**< 0.001**	**< 0.001**	**< 0.001**	**< 0.001**
R-Breast	Mean (Gy)	1.66 ± 0.54	1.90 ± 0.72	1.98 ± 0.59	0.77 ± 0.50	2.67 ± 0.85	0.051	0.051	**< 0.001**	**< 0.001**	**< 0.001**
Esophagus	Max (Gy)	36.75 ± 2.87	36.84 ± 2.66	37.78 ± 2.50	33.98 ± 4.40	38.35 ± 3.17	1.000	**0.01**	**0.049**	0.086	**0.001**
IDI_organs_		1.13 ± 1.55	1.43 ± 1.89	2.63 ± 3.22	2.91 ± 4.94	7.87 ± 15.69	0.924	0.097	0.575	0.499	0.617
PQI		12.30 ± 18.31	12.85 ± 16.51	31.79 ± 51.02	50.96 ± 86.84	67.55 ± 156.22	1.000	0.294	0.379	0.638	0.975
MU		848.43 ± 46.42	854.86 ± 65.35	808.29 ± 65.13	793.36 ± 54.35	876.45 ± 75.58	0.951	0.051	0.142	0.83	**0.011**

The left lung parameters, as compared to 3DCRT, V12Gy, V10Gy, V5Gy and the Mean dose in VMAT increased by 27.9%, 34%, 48.7%, and 20% respectively with *p* < 0.05, and similarly as compared to h-VMAT 80/20 by 23%, 21.2%, 12.5% and 11.4%. V20Gy, V30Gy were found less in hybrid plans of ratio 80/20 (18.51 ± 2.93%,9.97 ± 2.06%) and 70/30 (18.28 ± 2.80%,9.70 ± 1.80%), whereas V30Gy was found high in 3DCRT (13.28 ± 3.97%). The dosimetric results of the heart were comparatively less in h-VMAT 80/20 than all other plans; however, they obtained slightly higher than the clinical goal. The difference in heart parameters of h-VMAT 80/20, compared to 3DCRT was 2.28% in V25Gy and compared to VMAT 0.8 Gy in mean dose, 2.5% and 4.8% in V10Gy and V5Gy respectively with *p* < 0.05. The maximum heart dose was significantly higher in 3DCRT (37 Gy) than VMAT as well as hybrid plans (34 Gy). Similarly, 3DCRT resulted in increased maximum (35.14 ± 7.63) and mean dose (17.88 ± 7.46) to LAD and were comparatively less in h-VMAT 80/20 (28.89 ± 9.79 & 12.92 ± 6.40 respectively). There was no significant difference with h-VMAT 70/30, however h-VMAT 60/40 showed significant differences in mean dose, V10Gy, and V5Gy with *p* < 0.05. As an exception, the low dose spill of 2 Gy received by heart was least in 3DCRT (22.63 ± 6.90%; *p* < 0.05). The average mean dose of the right breast (0.77 ± 0.50 Gy) and lung (0.42 ± 0.11 Gy) were extremely significant, and the esophagus maximum dose (33.98 ± 4.40) resulted in the least in 3DCRT and was observed higher in VMAT. Hybrid ratio of 60/40 showed a trend of increase in all OAR dosimetric parameters compared to the other hybrid ratios.

h-VMAT 80/20 resulted in the lowest PQI scoring of 12.30 ± 18.31 in comparison with 3DCRT (50.96 ± 86.84) and VMAT (67.55 ± 156.22). Further, among the 3 ratios of hybrid, h-VMAT70/30 scored 12.85 ± 16.51, which was slightly higher than h-VMAT80/20, and h-VMAT60/40 scored 31.79 ± 51.02. In addition, the IDI_Tumor_ score was lower for VMAT(*p* < 0.005) than other plans; however, it resulted in a high IDI_Organs_ score of 7.87 ± 15.69. The MU was less in 3DCRT (793.36 ± 54.35) compared to both hybrid and VMAT plans, with a significance of *p* < 0.05 with VMAT.

## Discussion

Since studies evaluating the combination of hybrid and DIBH techniques for hypo-fractionated left breast RT are rare, our goal was to determine if DIBH, along with hybrid offers any further benefits in reducing OAR doses. Hence this dosimetric study aimed to explore the role of h-VMAT in FB and breath-hold conditions by comparing it with 3DCRT and VMAT techniques. Three different dose ratios of h-VMAT were also evaluated to find the optimum ratio.

Available articles on the hybrid techniques for breast RT have studied the combinations of 3DCRT&VMAT, 3DCRT&IMRT, and IMRT&VMAT. Most of them have used 3DCRT as the base dose plan with 70 to 80% contribution and the remaining with IMRT or VMAT plan to achieve better dosimetric results. Balaji et al. in their study, reported that the h-VMAT of 3DCRT(80%) and VMAT(20%) dose weightage reduces the uncertainty of dose delivery^21^. Similarly, Sathyaraj et al. also compared h-VMAT with VMAT for left breast RT using MONACO TPS^25^. Both the studies have shown the advantage of h-VMAT with a dose ratio of 80 − 70% from 3DCRT and 20–30% dose ratio from VMAT is optimum enough in obtaining the maximum benefit of h-VMAT over VMAT in left-sided CW irradiation with nodal involvement for conventional dose fractionation of 50 Gy in 25 fractions. Ramasubramianian et al. studied the various arc designs of h-VMAT for whole-breast RT and suggested two partial arc designs successfully achieved the planning goal with lower MU representing lesser beam-on time^22^. Moreover, it was also reported that partial arc designs are associated with better conformity and homogeneity at the expense of lower OAR dose than quarter arc designs. Studies comparing h-VMAT over a hybrid of 3DCRT&IMRT and IMRT&VMAT obtained higher MU in the later combinations resulting in increased treatment time^20,26^. Most of the studies concluded higher MU in IMRT increases the treatment uncertainty and poses a high risk of secondary cancer, in contrast, a recent study proposed a higher projected secondary cancer risk ratio from h-VMAT in contralateral organs compared to hybrid IMRT^27^. Considering the prolonged treatment planning time and higher uncertainty in treatment delivery of IMRT, our hybrid approach opted for a combination of 3DCRT& half arc VMAT plans to achieve higher target dose conformity in both BCS and mastectomy as well. Furthermore, wedges were not included in the 3DCRT plan as wedges contribute to higher MU^28^. Though the monitor units were not considerably reduced in hybrid approach of the present study, the possibilities of secondary cancer risk may be similar as that of 3DCRT plan than VMAT since the 3DCRT portal contributes 70–80% of dose than the dose modulation obtained from VMAT.

Exposure of heart volume needs to be kept low to minimize the risk of radiation-induced cardiac events post-RT; therefore, hybrid techniques aimed to minimize the high and low dose received volume of heart. It is well known that integrating the breath-hold method with the RT technique is beneficial in reducing high dose received heart volume as the distance of the heart from the chest wall increases^29^. In our study, an average 6 mm heart displacement from the breath-hold method reduced the mean heart dose by 1 Gy compared to the FB methods of the same planning technique. This significant difference was observed in all techniques (*p* < 0.001). A minimum heart dose of < 3 Gy was achieved for the h-VMAT80/20 plan under the DIBH method compared to 3DCRT and VMAT plans. In h-VMAT 80/20, V25Gy representing a high dose volume of heart was reduced to 1.34% in DIBH and comparatively less than all other plans. Yeh et al. in their h-VMAT study for early-stage breast RT, reported that a heart displacement above 3 cm in DIBH can reduce the heart mean dose by 2Gy^30^. Studies have reported significance dose reduction in LAD region in DIBH than FB method. Ferdinand et al., reported that the DIBH-3DCRT plan showed a 19% reduction in maximum dose to LAD^31^which is 22% in h-VMAT 80/20 plan of our study and mean dose to LAD was reduced by 6 Gy which was reported 8 Gy reduction by Sakyanun et al.^32^. As it is known that V20Gy, mean dose, V10Gy and V5Gy of the lung are correlated with radiation pneumonitis^33,34^increased volume of the lung from expansion during DIBH is also helpful in reducing lung exposure. In our study, the left lung receiving V30Gy and V20Gy were comparatively less in h-VMAT80/20 under the DIBH method and significantly less than FB. It is observed that the average V12Gy of left lung (approximately representing V10Gy) found to be well within the planning objective in all the plans except for VMAT. In addition, V2Gy of the heart showed a significant reduction (*p* < 0.001) in all FB plans and V5Gy of the Left lung representing low dose were found lowest in 3DCRT. Further, low dose spill to healthy tissue was also reduced in hybrid plans as the mean dose to the right breast and right lung were reduced compared to VMAT plans. h-VMAT plans yielded slightly less MU than VMAT, which might reduce the treatment time and increase patient comfort since breath-hold RT is time-consuming.

To choose the optimum dose ratio of h-VMAT, we have opted for a mathematical formula-based PQI scoring method on dosimetric parameters rather than ranking-based scoring as used in the previous study^18^. This formula is effective in evaluating the plans by collectively accounting for the deviation of target and organs from the planning criteria^22^. According to that, in our study, h-VMAT 80/20 and h-VMAT 70/30 were found effective in scoring minimum PQI compared to h-VMAT 60/40. In addition, 3DCRT and VMAT scored higher PQI values compared to all hybrid combinations. It is observed that these two h-VMAT ratios successfully reduced the volume of OAR receiving a high dose compared to 3DCRT and a low dose compared to VMAT. For patients with the ability to execute voluntary DIBH, h-VMAT plans may be highly advantageous as OAR doses are reduced with acceptable PTV coverage, which may result in increased quality of life. As the hybrid technique is a combination of two different techniques, future research requires measurement of the scattered dose to out-of-field regions from both techniques using appropriate detectors.

The limitations include the use of half arcs and not testing for quarter arcs in both h-VMAT and VMAT. In addition, impact of DIBH with h-VAMT on a subset of patients, such as Intramammary node(IMN) involvement and node-negative cases, has not been studied. And also, the practical implications such as difficulties in application, executing DIBH, reproducibility and set up errors were not studied.

We conclude that the h-VMAT technique in left breast RT for both BCS and mastectomy offers comparable dose conformity and homogeneity of the target as that of VMAT. For OARs, it reduces the low dose received volume compared to VMAT and the high dose received volume compared to 3DCRT (heart and left lung). Integrating DIBH with h-VMAT, especially h-VMAT 80/20 & 70/30 dose ratio, observed a further reduction of heart and lung dose, contributing to improved plan quality compared to 3DCRT and VMAT.

## Data Availability

The data of this study is provided within this manuscript.
